# Correction: Magin et al. Impact of Non-Covalent Interactions of Chiral Linked Systems in Solution on Photoinduced Electron Transfer Efficiency. *Int. J. Mol. Sci.* 2023, *24*, 9296

**DOI:** 10.3390/ijms26020433

**Published:** 2025-01-07

**Authors:** Ilya M. Magin, Ivan A. Pushkin, Aleksandra A. Ageeva, Sofia O. Martianova, Nikolay E. Polyakov, Alexander B. Doktorov, Tatyana V. Leshina

**Affiliations:** 1 Voevodsky Institute of Chemical Kinetics and Combustion, 630090 Novosibirsk, Russia; magin@kinetics.nsc.ru (I.M.M.); pushkin@kinetics.nsc.ru (I.A.P.); al.ageeva@gmail.com (A.A.A.); smartle636@gmail.com (S.O.M.); polyakov@kinetics.nsc.ru (N.E.P.); leshina@kinetics.nsc.ru (T.V.L.); 2 Department of Natural Sciences, Novosibirsk State University, 630090 Novosibirsk, Russia

The journal’s Editorial Office and Editorial Board are jointly issuing a resolution and removal of the Journal Notice linked to this article [1], as well as an update to the original publication. Following concerns raised about the integrity of the peer review, the Editorial Office has conducted a post-publication peer review of this article. This process included the recruitment of a new independent reviewer and was supervised by the original Academic Editor to ensure full compliance with MDPI’s Editorial Process (https://www.mdpi.com/editorial_process).

As a result of this process, the Academic Editor and the authors have agreed to update the following aspect of this publication:

- Reviewer Report 1 was removed from the peer review record and the new report was uploaded instead (https://www.mdpi.com/1422-0067/24/11/9296/review_report).

Based on the new review report, the following corrections have been made to the original publication:

The Keywords and Materials and Methods sections have been revised and Figures 1–8, Schemes 1 and 2 were corrected. 

With this update, the Academic Editor is satisfied that the Editorial Process relating to this article has been completed as per MDPI’s Editorial Process policy. The Editorial Office would like to thank the authors for their collaboration during this process. 
