# Supplementary material for: Impact of Non-Covalent Interactions of Chiral Linked Systems in Solution on Photoinduced Electron Transfer Efficiency
Source: Int J Mol Sci. 2023 May 26;24(11):9296. doi: 10.3390/ijms24119296 (PMC10253034; doi:10.3390/ijms24119296)
Supplement: Supplementary file 1 [file ijms-24-09296-s001.zip › ijms-2397602-supplementary.pdf]

SUPPLEMENTARY MATERIALS

Impact of Non-Covalent Interactions of Chiral Linked Systems in Solution on Photoinduced Electron Transfer Efficiency

Ilya M. Magin[1], Ivan A. Pushkin[1,2], Aleksandra A. Ageeva[1,2], Sofia O. Martianova[1,2], Nikolay E. Polyakov[1], Alexander B. Doktorov[1*] and Tatyana V. Leshina[1]

[1]Voevodsky Institute of Chemical Kinetics and Combustion, 630090 Novosibirsk, Russia;

[2]Department of Natural Sciences, Novosibirsk State University, 630090 Novosibirsk, Russia

* Correspondence: doktorov@kinetics.nsc.ru

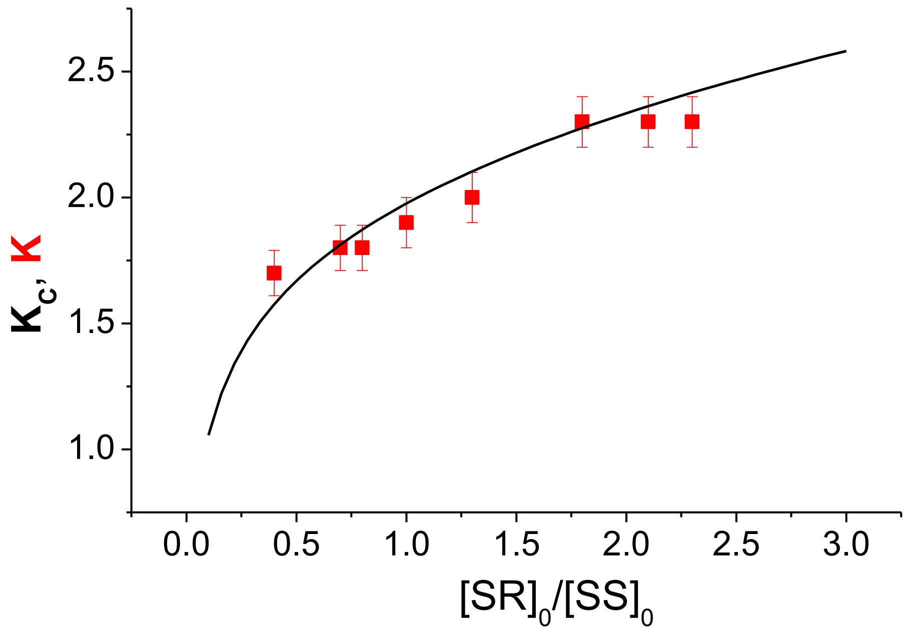

Figure S1. Experimental (**K**) and calculated (**K_C**) dependences of the CIDNP on the diastereomers (R,S)NPX-Pyr dyad concentrations ratio. Calculation parameters: α_{SR}=2, α_{SS}=1, β_{SR}=0.1, β_{SS}=0.1, γ=0; dimerization equilibrium constants: $K_{RS}=2\times10^5$ M^{-1}, $K_{SS}=2\times10^5$ M^{-1}, $K_{RS,SS}=1\times10^5$ M^{-1}. Here, α, β and γ are the efficiencies of polarization formation in homo-, hetero-dimers and monomers.

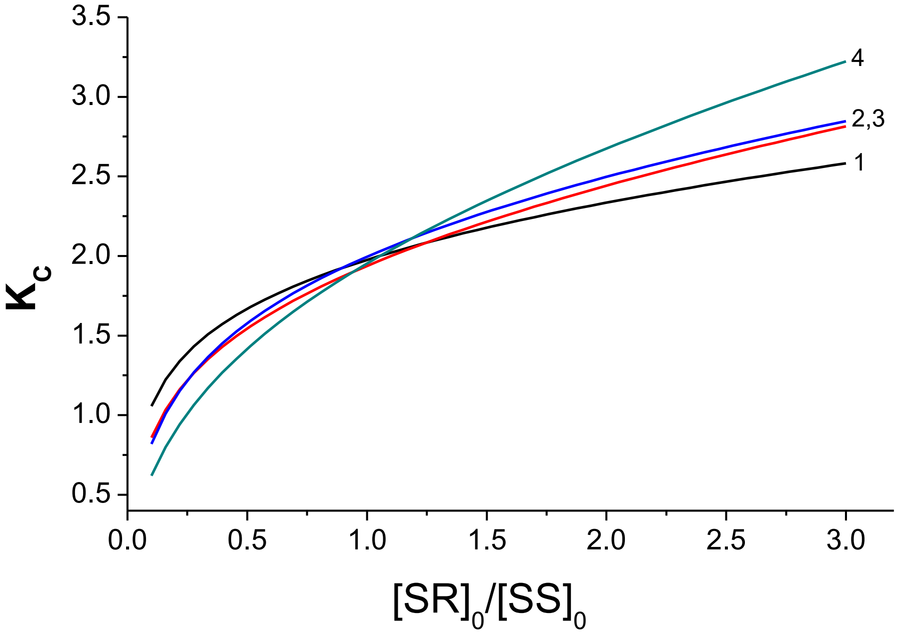

Figure S2. Calculated (**K_c**) dependences of the CIDNP on the diastereomers (R,S)NPX-Pyr dyad concentrations ratio with different dimerization equilibrium constants. Calculation parameters: $\alpha_{SR}=2$, $\alpha_{SR}=1$, $\beta_{SR}=0.1$, $\beta_{SS}=0.1$, $\gamma=0$. Dimerization equilibrium constants: 1) $K_{SR,SR}=2\times10^5$ M^{-1}, $K_{SS,SS}=2\times10^5$ M^{-1}, $K_{SR,SS}=1\times10^5$ M^{-1}; 2) $K_{SR,SR}=1\times10^5$ M^{-1}, $K_{SS,SS}=2\times10^5$ M^{-1}, $K_{SR,SS}=1\times10^5$ M^{-1}; 3) $K_{SR,SR}=2\times10^5$ M^{-1}, $K_{SS,SS}=1\times10^5$ M^{-1}, $K_{SR,SS}=1\times10^5$ M^{-1}; 4) $K_{SR,SR}=2\times10^5$ M^{-1}, $K_{SS,SS}=2\times10^5$ M^{-1}, $K_{SR,SS}=2\times10^5$ M^{-1}. Here, α, β and γ are the efficiencies of polarization formation in homo-, hetero-dimers and monomers.

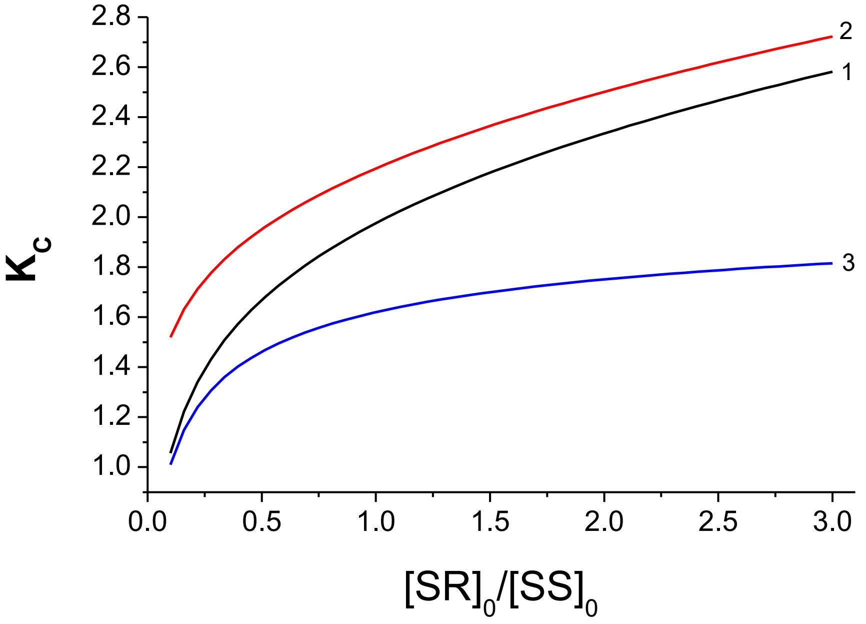

Figure S3. Calculated (**K_c**) dependences of the CIDNP on the diastereomers (R,S)NPX-Pyr dyad concentrations ratio with different β. Calculation parameters: α_{SR}=1.8, α_{SS}=1, γ=0; dimerization equilibrium constants: $K_{RS}=2\times10^5$ M^{-1}, $K_{SS}=2\times10^5$ M^{-1}, $K_{RS,SS}$ = 1×10^5 M^{-1}. 1) β_{SR}=0.1, β_{SS}=1; 2) β_{SR}=0.1, β_{SS}=1, 3) β_{SR}=0.1, β_{SS}=1; Here, α, β and γ are the efficiencies of polarization formation in homo-, hetero-dimers and monomers.

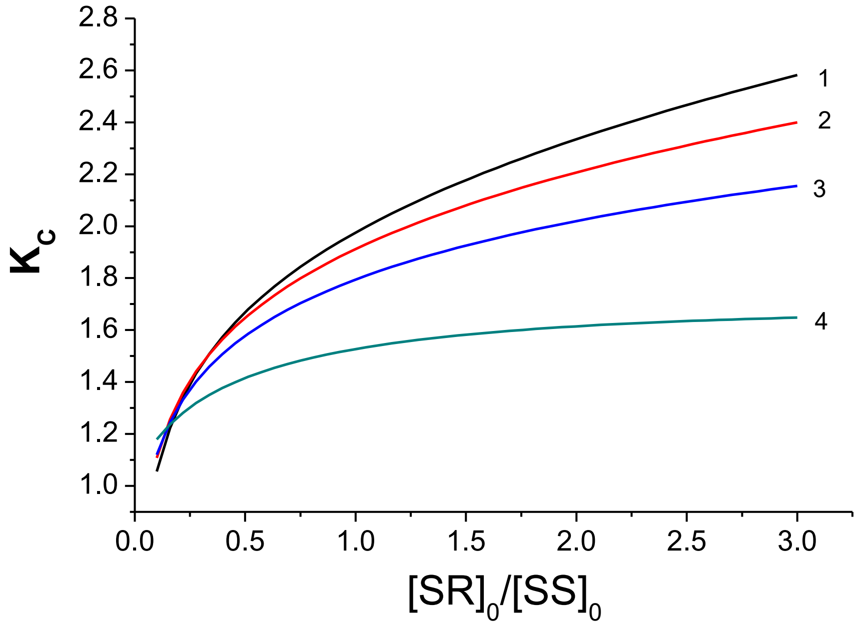

Figure S4. Calculated (**K_c**) dependences of the CIDNP on the diastereomers (R,S)NPX-Pyr dyad concentrations ratio with different dimerization equilibrium constants, taking into account γ. Calculation parameters: α_{SR}=2, α_{SR}=1, β_{SR}=0.1, β_{SS}=0.1. Dimerization equilibrium constants: 1) γ=0, $K_{SR,SR}=2\times10^5$, $K_{SS,SS}=2\times10^5$, $K_{SR,SS}=1\times10^5$ M^{-1}; 2) γ=1, $K_{SR,SR}=2\times10^5$ M^{-1}, $K_{SS,SS}=2\times10^5$ M^{-1}, $K_{SR,SS}=1\times10^5$ M^{-1}; 3) γ=1, $K_{SR,SR}=2\times10^4$ M^{-1}, $K_{SS,SS}=2\times10^4$ M^{-1}, $K_{SR,SS}=1\times10^4$ M^{-1}; 4) γ=1, $K_{SR,SR}=2\times10^3$ M^{-1}, $K_{SS,SS}=2\times10^3$ M^{-1}, $K_{SR,SS}=1\times10^3$ M^{-1}. Here, α, β and γ are the efficiencies of polarization formation in homo-, hetero-dimers and monomers.

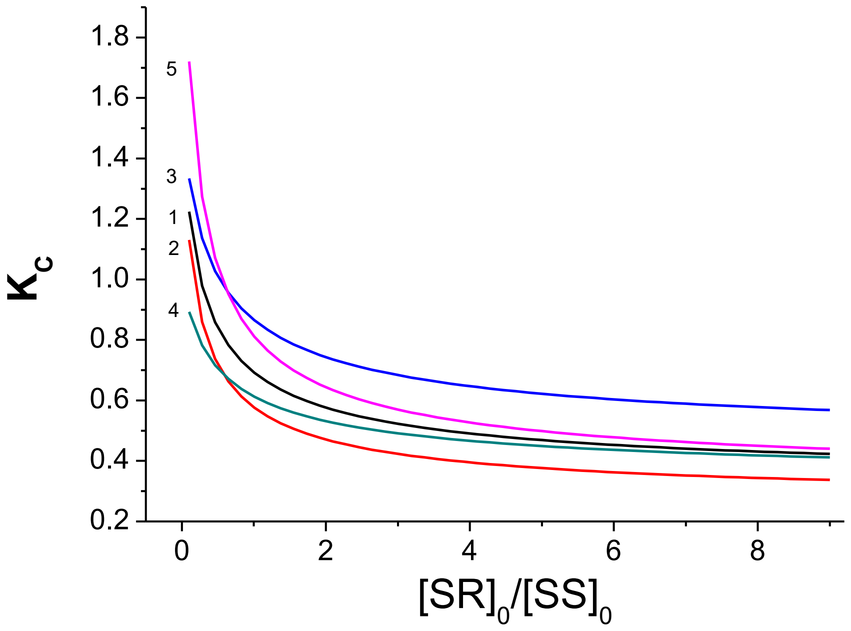

Figure S5. Calculated ($\mathbf{K_c}$) dependences of the CIDNP on the diastereomers (R,S)NPX-Trp dyad concentrations ratio with different β. Calculation parameters: α_{SR}=1.8, α_{SS}=1, γ=0; dimerization equilibrium constants: K_{RS} = 2×10^5 M^{-1}, K_{SS} = 2×10^5 M^{-1}, $K_{RS,SS}$ = 2×10^5 M^{-1}. 1) β_{SR}=1.6, β_{SS}=5.5; 2) β_{SR}=1.6, β_{SS}=7; 3) β_{SR}=1.6, β_{SS}=4; 4) β_{SR}=1, β_{SS}=5.5; 5) β_{SR}=2.5, β_{SS}=5.5. Here, α, β and γ are the efficiencies of polarization formation in homo-, hetero-dimers and monomers.

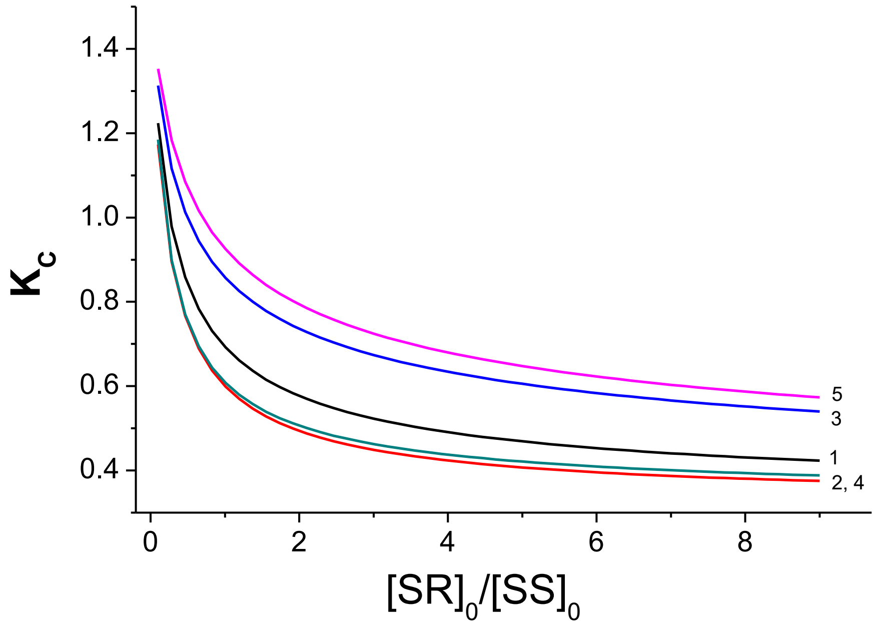

Figure S6. Calculated ($\mathbf{K_c}$) dependences of the CIDNP on the diastereomers (R,S)NPX-Trp dyad concentrations ratio with different dimerization equilibrium constants. Calculation parameters: α_{SR}=1.8, α_{SR}=1, β_{SR}=1.6, β_{SS}=5.5, γ=0.

Dimerization equilibrium constants: 1) $K_{SR,SR}=2\times10^5$ M^{-1}, $K_{SS,SS}=2\times10^5$ M^{-1}, $K_{SR,SS}=2\times10^5$ M^{-1}; 2) $K_{SR,SR}=1\times10^5$, $K_{SS,SS}=2\times10^5$ M^{-1}, $K_{SR,SS}=2\times10^5$ M^{-1}; 3) $K_{SR,SR}=5\times10^5$ M^{-1}, $K_{SS,SS}=2\times10^5$ M^{-1}, $K_{SR,SS}=2\times10^5$ M^{-1}; 4) $K_{SR,SR}=2\times10^5$ M^{-1}, $K_{SS,SS}=1\times10^5$ M^{-1}, $K_{SR,SS}=2\times10^5$ M^{-1}; 5) $K_{SR,SR}=2\times10^5$ M^{-1}, $K_{SS,SS}=2\times10^5$ M^{-1}, $K_{SR,SS}=1\times10^5$ M^{-1}. Here, α, β and γ are the efficiencies of polarization formation in homo-, hetero-dimers and monomers.

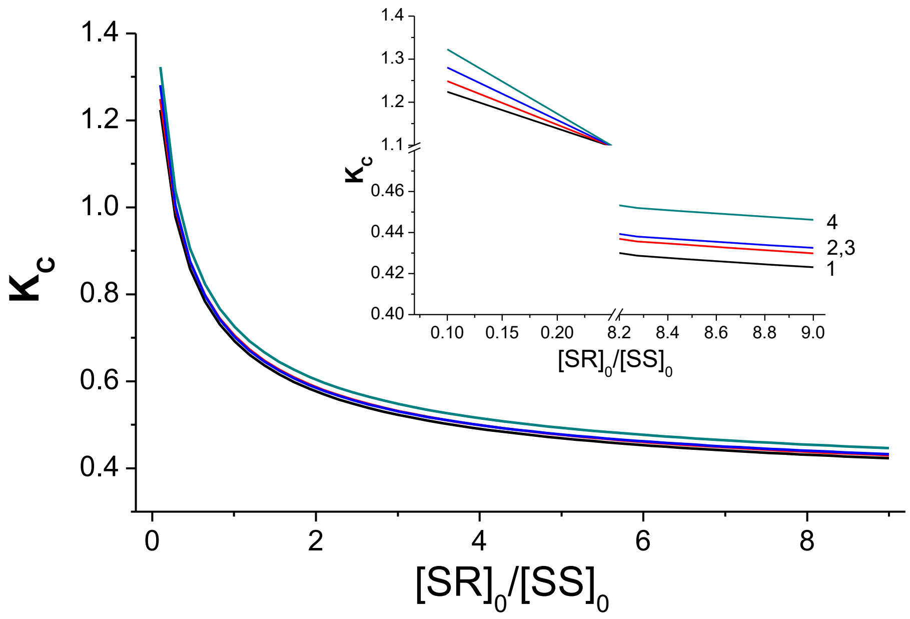

Figure S7. Calculated (**K_c**) dependences of the CIDNP on the diastereomers (R,S)NPX-Trp dyad concentrations ratio with different γ at height dimerization equilibrium constants. Calculation parameters: $\alpha_{SR}=1.8$, $\alpha_{SR}=1$, $\beta_{SR}=1.6$, $\beta_{SS}=5.5$. Dimerization equilibrium constants γ. $K_{SR,SR}=2\times10^5$ M^{-1}, $K_{SS,SS}=2\times10^5$ M^{-1}, $K_{SR,SS}=2\times10^5$ M^{-1} 1) γ=0; 2) γ=2; 3) γ =5; 4) γ = 5. Here, α, β and γ are the efficiencies of polarization formation in homo-, hetero-dimers and monomers

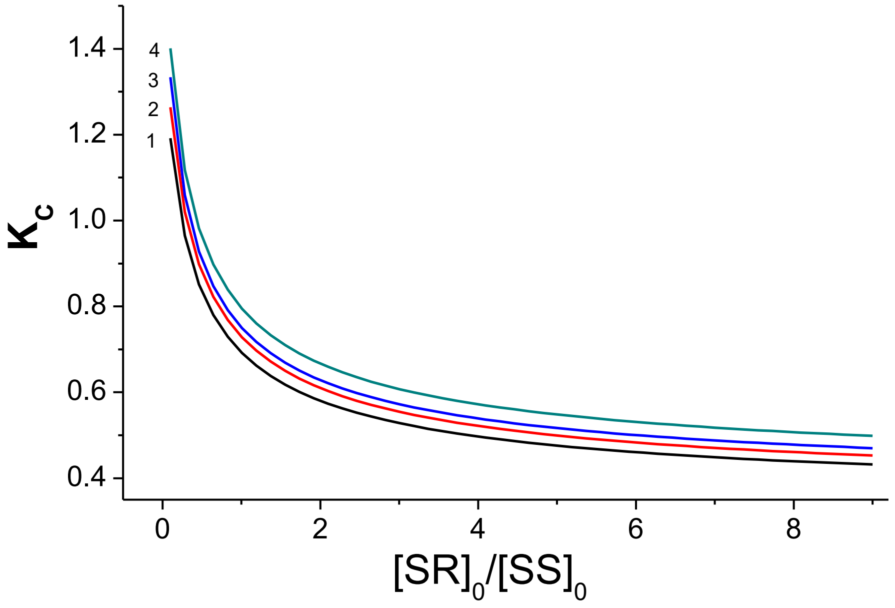

Figure S8. Calculated (**K_c**) dependences of the CIDNP on the diastereomers (R,S)NPX-Trp dyad concentrations ratio with different γ at medium dimerization equilibrium constants. Calculation parameters: α_{SR}=1.8, α_{SR}=1, β_{SR}=1.6, β_{SS}=5.5. Dimerization equilibrium constants γ. $K_{SR,SR}=2\times10^4$ M^{-1}, $K_{SS,SS}=2\times10^4$ M^{-1}, $K_{SR,SS}=2\times10^4$ M^{-1} 1) γ=0; 2) γ=2; 3) γ=5; 4) γ=10. Here, α, β and γ are the efficiencies of polarization formation in homo-, hetero-dimers and monomers.

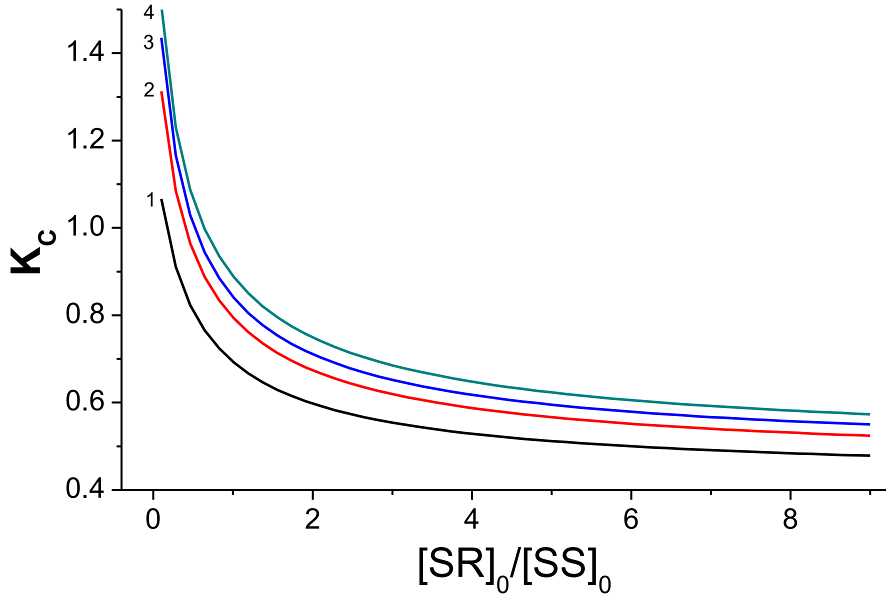

Figure S9. Calculated (**K_c**) dependences of the CIDNP on the diastereomers (R,S)NPX-Trp dyad concentrations ratio with different γ at low dimerization equilibrium constants. Calculation parameters: α_{SR}=1.8, α_{SR}=1, β_{SR}=1.6, β_{SS}=5.5.

Dimerization equilibrium constants γ. $K_{SR,SR}=2\times10^3$, $K_{SS,SS}=2\times10^3$, $K_{SR,SS}=2\times10^3$. 1) $\gamma=0$; 2) $\gamma=2$; 3) $\gamma=5$; 4) $\gamma=10$. Here, α, β and γ are the efficiencies of polarization formation in homo-, hetero-dimers and monomers.
